# Identification of novel genes including *NAV2* associated with isolated tall stature

**DOI:** 10.3389/fendo.2023.1258313

**Published:** 2023-12-12

**Authors:** Birgit Weiss, Tim Ott, Philipp Vick, Julian C. Lui, Ralph Roeth, Sebastian Vogel, Stephan Waldmüller, Sandra Hoffmann, Jeffrey Baron, Jan M. Wit, Gudrun A. Rappold

**Affiliations:** ^1^ Institute of Human Genetics, Heidelberg University, Heidelberg, Germany; ^2^ Department of Zoology, University of Hohenheim, Stuttgart, Germany; ^3^ National Institute of Child Health and Human Development, National Institutes of Health, Bethesda, MD, United States; ^4^ Institute of Medical Genetics and Applied Genomics, University of Tübingen, Tübingen, Germany; ^5^ Division of Pediatric Endocrinology, Department of Pediatrics, Willem-Alexander Children’s Hospital, Leiden University Medical Center, Leiden, Netherlands

**Keywords:** isolated tall stature, growth plate, NAV2, all-trans retinoic acid, oligogenic inheritance, IFT140, Xenopus

## Abstract

Very tall people attract much attention and represent a clinically and genetically heterogenous group of individuals. Identifying the genetic etiology can provide important insights into the molecular mechanisms regulating linear growth. We studied a three-generation pedigree with five isolated (non-syndromic) tall members and one individual with normal stature by whole exome sequencing; the tallest man had a height of 211 cm. Six heterozygous gene variants predicted as damaging were shared among the four genetically related tall individuals and not present in a family member with normal height. To gain insight into the putative role of these candidate genes in bone growth, we assessed the transcriptome of murine growth plate by microarray and RNA Seq. Two (*Ift140, Nav2*) of the six genes were well-expressed in the growth plate. *Nav2* (*p*-value 1.91E-62) as well as *Ift140* (*p*-value of 2.98E-06) showed significant downregulation of gene expression between the proliferative and hypertrophic zone, suggesting that these genes may be involved in the regulation of chondrocyte proliferation and/or hypertrophic differentiation. *IFT140, NAV2* and *SCAF11* have also significantly associated with height in GWAS studies. Pathway and network analysis indicated functional connections between *IFT140*, *NAV2* and *SCAF11* and previously associated (tall) stature genes. Knockout of the all-trans retinoic acid responsive gene, neuron navigator 2 *NAV2*, in *Xenopus* supports its functional role as a growth promotor. Collectively, our data expand the spectrum of genes with a putative role in tall stature phenotypes and, among other genes, highlight *NAV2* as an interesting gene to this phenotype.

## Introduction

Tall stature, by definition, comprises the upper 2.3 percent of a population (1). Pathological causes of tall stature are rare and include endocrine diseases (such as GH producing tumours) and various genetic conditions, such as chromosomal abnormalities (e.g., Klinefelter syndrome), monogenic conditions (Marfan (MIM #154700), Sotos (MIM #117550) or Weaver (MIM #277590) syndromes) or imprinting disorders (Beckwith-Wiedemann syndrome) (1, 2). In most cases, isolated (non-dysmorphic) tall stature runs in the family and gets the diagnostic label of familial or constitutional tall stature. Since no cause can be established in such cases, we suggested to use the term “idiopathic tall stature”, with two subgroups (familial and non-familial) (1).

Individuals with non-syndromic (idiopathic) tall stature that seek advice in a clinic, often remain undiagnosed. When genetic evaluations are being performed in these individuals, monogenic causes cannot be found in the great majority of cases, suggesting that oligogenic effects may underlie this phenotype. Isolated tall stature therefore remains a challenge for clinicians and geneticists alike. Several thousands of genes have been associated with height in Genome-wide association studies (GWAS) (3), but only a small number of those are considered of definitive diagnostic value for tall stature phenotypes. Examples of genes with diagnostic value comprise duplications of the *SHOX* and IGF1R genes and cases carrying an activating *NPR2* mutation (4–8).

Here, we studied a three-generation family with extreme tall stature, and aimed to identify genetic variants shared by the tall members of this family. For this purpose, sequence variants in the tall individuals were analysed by whole exome sequencing and compared with data from a control cohort. Expression analysis in the growth plate and genome-wide association studies (GWAS) furthermore supported a causative role for three particular genes in tall stature. The strongest candidate gene was analysed in the amphibian model *Xenopus.*

## Materials and methods

### Clinical height data

Our index case, individual II.1, was extremely tall in adolescence and treated with high dose testosterone esters. At 56 years of age, we measured his height at 208.6 cm, which would translate to 211 cm (4.0 SDS) at 21 years (due to shrinking with age) (9). Yet, this may still be an underestimation in view of the treatment with testosterone esters in adolescence. Sitting height was 104 cm (sitting height/height -1.0 SDS) (10) and head circumference 60 cm (1.3 SDS) (11). He was married to a tall woman (II.2; 3.2 SDS) and had a tall son (III.1; 2.8 SDS). His sister (II.4; 3.2 SDS) married a man with normal height (II.3; 1.7 SDS) and had a daughter of normal height (III.2; -0.7 SDS). Height data were adjusted for secular trend and shrinking (9). Subjects II.1, II.4 and III.1 were examined in childhood and adolescence by experienced paediatric endocrinologists, who documented a stable height SDS and an absence of any signs of growth overproduction or dysmorphic syndrome. Further information on subjects and growth measurements, and on the calculation of adjusted height and shrinking, is given in the **Supplementary Information**.

Candidate genes identified in this study were also checked in all tall family members that we previously described in Weiss et al, 2021 (12).

### Exome sequencing and filtering

Exome sequencing was conducted on genomic DNA from peripheral blood leucocytes. In brief, coding genomic regions were enriched using the SureSelect XT Human All Exon Kit V7 (Agilent Technologies, Santa Clara, CA, USA) for subsequent sequencing as 2x125 or 2x100 bp paired-end reads on a HiSeq2500 or NovaSeq6000 system (Illumina, San Diego, CA, USA). Generated sequences were analysed using the megSAP pipeline (https://github.com/imgag/megSAP). Clinical variant prioritization included different filtering steps and was conducted either A) in a single-mode (MAF [minor allele frequency] in 1000g, ExAC, gnomAD or in an in-house database set to <0.1% [autosomal-dominant] or <1% [autosomal-recessive]) or B) in a multi-mode (prioritizing only those variants shared by family members with tall stature), with three different sub-types: multi-sample dominant, multi-sample compound-heterozygous [both with a MAF cut-off of <0.1%] and multi-sample recessive [MAF cut-off: <1%]).

### Sanger sequencing and analysis of variants

Variants of interest were confirmed by Sanger sequencing. Polymerase chain reaction (PCR) with primers indicated in **Supplementary Table 1** was performed with HotStar Taq Polymerase and products analysed on agarose gels and subsequently sequenced using an ABI machine (Azenta Life Sciences).

We also checked for duplications including SHOX and IGF1R duplications and XYY in all the exome data sets and can exclude complete duplications. Partial duplications of these genes are also unlikely, but we cannot totally exclude them due to methodological limitations. We also checked for IGF-1R variants in the exome data set and re-checked the three known NPR2 mutations from the literature that are associated with tall stature and found no such variants in this family.

Variants were also checked in the “normal Non-Finnish control cohort” from gnomAD, which does not specify the height of the control individuals. Therefore, it is possible that single gene variants can be present in controls at low allele frequencies.

### Network and pathway analysis

QIAGEN Ingenuity Pathway Analysis (IPA; https://digitalinsights.qiagen.com/) was applied to predict functional connections. IPA integrates selected omics data sets with mining techniques. Interpretation was in the context of protein networks that comprise protein-protein interactions and related biological functions and canonical signalling pathways.

### Databases

TGP (https://browser.1000genomes.org)**;** *GnomAD* (https://gnomad.broadinstitute.org/); *CADD score* (https://cadd.gs.washington.edu); dbSNP (https://www.ncbi.nlm.nih.gov); *GTEx database* (www.gtexportal.org); PROVEAN/SIFT (http://provean.jcvi.org/index.php); Polyphen2 (http://genetics.bwh.harvard.edu/pph2/); GWAS (www.ebi.ac.uk); Disease knowledge portal (https://cvd.hugeamp.org); IPA Ingenuity Systems (https://digitalinsights.qiagen.com/).

### Expression analysis in growth plates

Gene expression analysis in mouse growth plate was carried out to analyse (1) growth plate specificity: comparing mRNA expression in growth plate of 1-week-old mice with expression in three different soft tissues (lung, kidney and heart) using microarray (13) (2); spatial regulation: comparing mRNA expression in resting, proliferative and hypertrophic zones in the 1-week-old mouse growth plate by RNA-Seq; and (3) temporal regulation: comparing mRNA expression at 1 and 4 weeks of age in proliferative and hypertrophic zones of mouse growth plate by RNA-Seq (14). For spatial comparison, chondrocytes from different zones of 1-week-old mouse proximal tibia growth plate were isolated by laser capture microdissection as previously described (15). The spatial analysis of gene expression in the growth plate has previously been validated by demonstrating that it correctly identifies the spatial expression pattern of known growth plate zone markers (14). Sequencing libraries were prepared using a TruSeq Stranded mRNA Prep Kit (Illumina). Sequencing was performed via a paired end 75 cycle on Illumina HiSeq 2500. RNA-Seq reads were trimmed with cutadapt (-AAGATCGGAAGAGCACACGTCTGAACTCCAGTCAAAGATCGGAAGAGCGTCGTGTAGGGAAAGA GTGT -overlap 6 -q 20 -minimum-length 25) and aligned using STAR (2 pass alignment) to mouse mm10 reference genome sequences. Log2 fold changes and p-values (adjusted for multiple comparison using the Benjamini Hochberg correction) were generated by DESeq2.

### Functional studies in *Xenopus*

#### *Xenopus laevis* care and maintenance

*Xenopus laevis* was purchased from Nasco (901 Janesville Avenue, P.O. Box 901, Fort Atkinson, WI, USA). Handling, care and experimental manipulations of animals was approved by the Regional Government Stuttgart, Germany (V349/18 ZO) according to German regulations and laws (§6, article 1, sentence 2, nr. 4 of the animal protection act). Animals were kept at the appropriate conditions (pH=7.7, 20°C) in the animal facility. To induce ovulation, female frogs were injected subcutaneously with 300-700 units of human chorionic gonadotropin (Sigma). Individual embryos from one batch were randomly picked and used either as control specimens or for injections.

#### CRISPR/Cas9 sgRNA design and microinjections

Two sgRNA were designed using the publicly available ‘CRISPRscan’ software (16), each targeting a distinct exon in the orthologous *Xenopus laevis nav2* gene (with each sgRNA targeting the L- and S-forms simultaneously). sgRNA1 was designed to mutate the conserved exon corresponding to the human exon where the R2142C missense mutation is located, potentially inducing a truncated version of the Nav2 protein (5’- GCGCCAGTACCTCTCCCACG -3’). sgRNA2 was designed to target a large 5’-located exon that is conserved in all transcript variants (corresponding to the human amino acid sequence AIPQPGA) (5’- GCAGTTGGGCTGGGGGATGG -3’). sgRNAs were transcribed with the MEGAshortscript T7 Kit from synthetic DNA oligomers and purified with the MEGAclear Transcription Clean-Up Kit (both ThermoFisher). Oligos for synthesis were:

sgRNA1_GCAGCTAATACGACTCACTATAGGGCCAGTACCTCTCCCACGGTTTTAGAGCTAGAAATAGCAAG.

sgRNA2_GCAGCTAATACGACTCACTATAGGAGTTGGGCTGGGGGATGGGTTTTAGAGCTAGAAATAGCAAG.

Embryos were injected with 1 ng Cas9 protein (PNA Bio) and 300 pg sgRNA at 1-cell stage, embryos were then cultivated at 18°C until stage 45 and fixed for length analyses. DNA was isolated from 10 mutant or 5 control embryos. Un-injected littermates served as controls. PCR-based amplification for DNA samples was according to the manufacturer’s protocol, adapted for *taq* polymerase conditions. PCR oligos were the following: sgRNA1(L)-F_5’-AACGGGTTTCCGGCTAACTG-3’ and R_5’- GAGGTGGCTTGGTTCATGGT-3’, sgRNA1(S)-F_5’- TTCTTAGAAGCTGGTGGTGGT-3’ and R_5’- GGGGCTGGAATAAAGACAGGA-3’, sgRNA2(L)-F_5’-TTTTCCTGGTGCGTGTGAGT-3’ and R_5’- ACCCTTCTAGGGGACTTCCG-3’, sgRNA2(S)-F_5’-CACACAAGGCAGGGAATGTG-3’ and R_5’- GGAGTCCAACCTCTCACAGC-3’. For verification of mutagenesis, amplified target sequences were Sanger sequenced using the respective PCR oligos. Sequence results were analyzed for mutagenesis rates (percentage of Indels, i.e. insertion or deletion of bases) in the targeted exons using Synthego’s ICE software [https://ice.synthego.com, (17)] and/or TIDE (18). Indel percentages for sgRNA1 were 38% (L-form) and 32% (S-form), for sgRNA2 93% (L-form) and 75% (S-form).

#### Analysis of tadpole body length

Embryos were mildly fixed in 4% formaldehyde in MEM buffer (MOPS/EGTA/MgSO_4_) for 2hrs. For tadpole body length analyses, group pictures of all embryos were taken of each experiment at stage 44-45, using the same setup and magnification. Length was quantified by measuring distances from the mouth opening/cement gland (the most anterior head structures) to the tip of the tail (excluding the fin tissue) using the segmented line tool in ImageJ (19). Pixel to millimeter conversion was calibrated using a photographed millimeter scale. To ensure consistency of the measurements, a single observer performed these measurements in both controls and treated animals.

#### Statistical analysis

To be able to compile the data of the three different clutches, we normalized body lengths (in mm) of each single experiment to the median of its controls and then combined relative values of all three experiments. Using this approach, we found a statistically significant overall effect with Wilcoxon rank sum test (controls were normally distributed but not the treated animals for the dataset; p-value = 0.023).

## Results

### Identification of novel gene variants associated with tall stature

**Figure 1:**
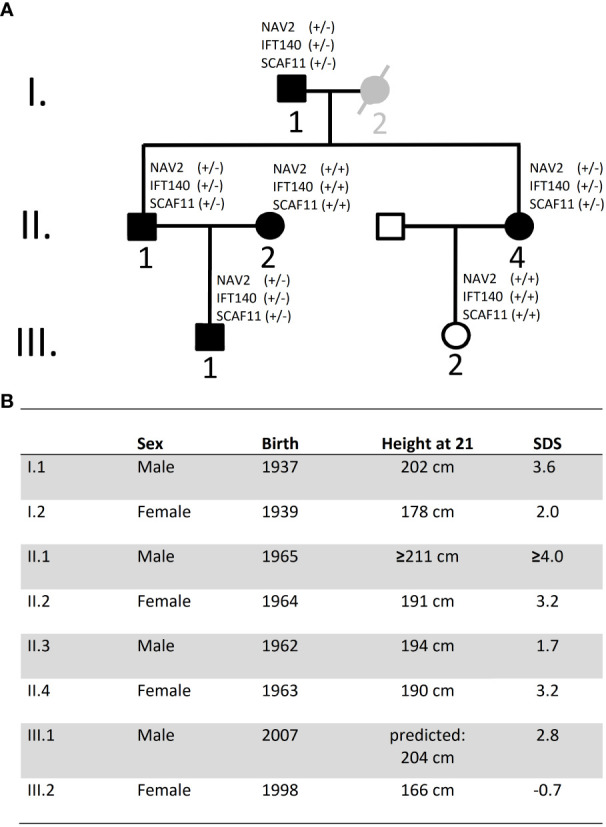
Pedigree and height data. **(A)** Pedigree of family: Black symbols indicate individuals with tall stature. Individual I.2 is considered as borderline tall stature (grey symbol). White symbols indicate normal stature. Exome sequencing was carried out on DNA from individuals I.1, II.1, II.4 and III.1. DNA was also available from I.2, II.2 and III.2 but not for exome sequencing; no DNA was available from individual II.3 (declined). **(B)** Data on height and year of birth of Family A Height data were adjusted for secular trend and shrinking. SDS, Standard deviation score.

Exome sequencing was carried out in the index case (individual II.1) and three additional tall members of a three-generation family (individuals I.1, II.4, III.1; **Figures 1A, B**). All individuals were healthy and without organomegaly nor dysmorphic features. Filtered variants predicted as damaging (based on Provean, SIFT and PPH2 scores) and not rated as polymorphisms (by gnomAD) and with high CADD scores, as well as shared among the related tall individuals, were first validated by Sanger sequencing and then checked for absence in a family member with normal stature (individual III.2, **Figure 1A**). Missense variants in the following six genes were classified as likely pathogenic*: CNGB1, GGTLC2, HSD3B2, IFT140, NAV2* and *SCAF11* (**Table 1**). Comparison to the normal Non-Finnish controls from gnomAD revealed allele frequencies of the individual variants between 10^-3^ to 10^-5^. The likelihood that a control individual carries several of these variants at the same time is minimal.

**Table 1:** Variants shared by tall individuals (I.1, II.1, III.1 and II.4) and not present in III.2 with normal height, considered damaging by at least one of the three prediction tools, and a CADD score above 15.

*Gene*	Chromosomal Position [GRCh38/hg38]	NT change	AA change	gnomAD		Number of homoz.	Allele frequency	CADD	PPH2	Provean	SIFT
				Allele count	Allele number						
*CNGB1*	chr16:57897524	c.3115G>A	p.G1039R	713	128476	1	5.4x10^-3^	26.6	probably damaging	deleterious	damaging
*GGTLC2*	chr22:22647025	c.347G>T	p.G116V	8	128498	0	9.4x10^-5^	20.2	probably damaging	deleterious	damaging
*HSD3B2*	chr1:119422001	c.500C>T	p.A167V	211	128918	0	1.6x10^-3^	16.8	possibly damaging	neutral	tolerated
*IFT140*	chr16:1511036	c.4297C>T	p.R1433C	170	121554	0	1.3x10^-3^	20.0	benign	deleterious	tolerated
*NAV2*	chr11:20103261	c.6424C>T	p.R2142C	513	117944	1	3.7.0x10^-3^	32.0	probably damaging	deleterious	damaging
*SCAF11*	chr12:45926753	c.2948G>A	p.R983Q	8	128952	0	4.7x10^-5^	25.3	probably damaging	neutral	damaging

All variants were missense variants. Only data of European Non-Finnish individuals (controls, gnomAD) was taken into account. AA, aminoacid; NT, nucleotide; CADD, combined annotation dependent depletion; PPH2, Provean and SIFT are prediction tools. The following Matched Annotation from NCBI and EMBL-EBI (MANE) Select transcripts were used as reference transcripts: CNGB1 (ENST00000251102.13):c.[3115G>A];[=], p.[(G1039R)];[(=)], GGTLC2 (ENST00000448514.3):[c.347G>T];[=], p.[(G116V)];[(=)], HSD3B2 (ENST00000369416.4):c.[500C>T];[=], p.[(A167V)];[(=)], IFT140 (ENST00000426508.7):c.[4297C>T]; [=], p.[(R1433C)];[(=)], NAV2 (ENST00000349880.9):c.[6424C>T];[=], p.[(R2142C)];[(=)], SCAF11 (ENST00000369367.8):c.[2948G>A];[=], p.[(R983Q)];[(=)].

*CNGB1 (cyclic nucleotide gated channel subunit beta 1)* encodes an ion channel protein; *GGTLC2 (gamma-glutamyltransferase light chain 2*) is involved in RNA binding and spliceosomal complex assembly; *HSD3B2* (*3-beta-hydroxysteroid dehydrogenase*) is essential for synthesis of multiple steroid hormones; *IFT140 (Intraflagellar transport 140 homolog)* is indispensable for intraflagellar transport in primary cilia; *NAV2* (*Neuron navigator 2)* is an all-trans retinoic acid responsive gene; and *SCAF11 (*SR-related CTD associated factor 11) encodes an RNA binding protein and is a regulator of spliceosome assembly.

From all affected amino acids of the six genes, species conservation was highest for *NAV2* (conserved between human and Drosophila) and lowest for *HSD3B2* (**Table 2**). *GTEx* expression analysis indicated that *CNGB1* is expressed in a brain-specific way, *GGTL2* in a testis-specific and *HSDB2* in an adrenal gland-specific manner, whereas expression of *IFT140, NAV2* and *SCAF11* is considered ubiquitous (**Supplementary Table 2**).

**Table 2:** Species conservation.

	CNGB1	GGTLC2	HSD3B2	IFT140	NAV2	SCAF11
**Human**	G	G	A	R	R	R
**Chimp.**	G	(–)	A	R	R	R
**Rhesus**	G	G	A	R	R	R
**Mouse**	G	(–)	A	R	R	R
**Chicken**	G	D	K	*na*	R	R
**Xenopus**	G	G	(–)	R	R	(–)
**Fugu**	(–)	(–)	L	(–)	R	(-)
**Zebrafish**	G	(-)	Q	R	R	G
**Drosophila**	A	(-)	N	*na*	R	Q
**C. elegans**	N	*na*	(-)	*na*	L	(-)

Capital letters indicate aminoacids (–);, indicates no homologue; na, indicates no alignment. Strongest conservation is seen for NAV2.

### Association with height by GWAS

We next determined whether the identified six genes were associated with height in GWAS studies using the Disease knowledge portal (https://cvd.hugeamp.org). We found *IFT140, NAV2* and *SCAF11* to be associated with height with genome-wide significance *(P*-values between 10^-11^ and 10^-14^), while no associations were reported for *CNGB1, GGTLC2* and *HSD3B2* (**Supplementary Table 3**). Because it is unknown whether common genetic variants (SNPs) associated with height are concentrated around the same genes where rare variants are identified, we decided that further evidence was needed.

### Network analysis

**Figure 2:**
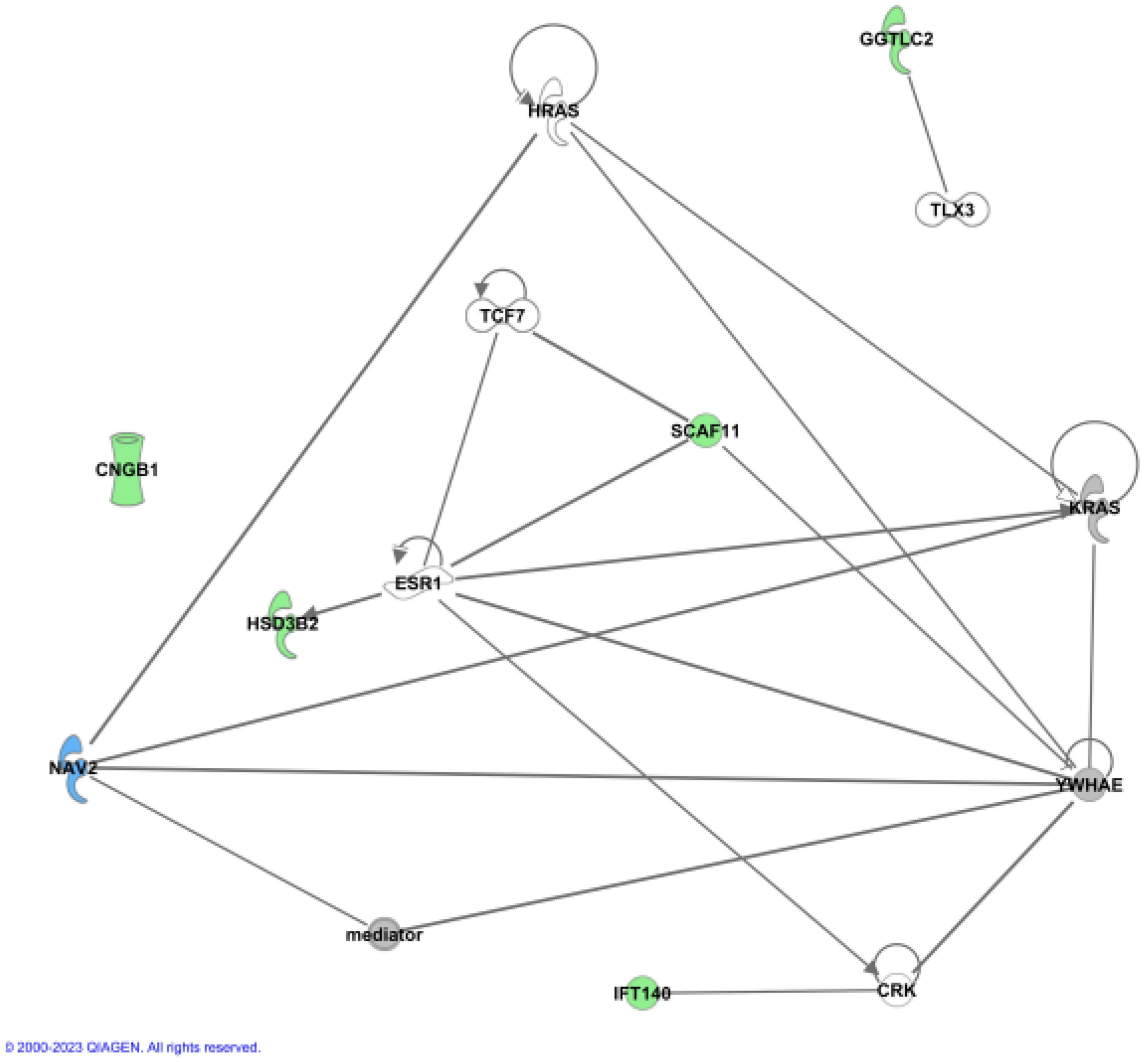
Ingenuity (IPA) Network analysis. The newly identified genes were used to show functional connections in context of known protein networks, based on data from the literature. No connections to known proteins could be established for CNGB1.

To further address the biological relevance of the identified gene variants, network analysis by Ingenuity Pathway Analysis (IPA) was carried out. IPA was used on the six novel candidate genes for tall stature together with 86 genes previously associated with tall stature by GWAS and literature data (**Supplementary Table 4**) (12, 20, 21). The newly identified six candidate genes were analysed to assess whether functional connections exist with known protein networks (**Figure 2** **;** **Supplementary Figure 1**). Our network analysis indicated direct protein-protein interactions between GGTLC2 and TLX3 (22), between IFT140 and CRK (23), and between SCAF11 and YWHAE (24) (**Figure 2**). Protein-protein interactions also exist between SCAF11 and the estrogen receptor ESR1 and the transcription factor TCF7 (25), and between HSD3B2 and ESR1 (26) (**Figure 2**). The all-trans retinoic acid-responsive NAV2 protein interacts with YWHAE, KRAS, HRAS and the multiprotein complex Vitamin D receptor-interacting protein mediator/TRAP (for thyroid hormone receptor-associated protein), which is highly associated with height (3.79E-28) (27–29). TRAP interacts with various transcription factors and acts as a transcriptional co-activator. CNGB1 did not interact with any of the known tall stature- associated genes.

Together, these data provide experimentally-based evidence that five of the six candidate genes are involved in protein-protein interactions with other known genes previously associated with height.

### Expression in the growth plate

To gain insight into the functional role of these candidate genes in bone growth, we assessed mRNA expression in murine growth plates. Growth plates are the cartilaginous structures residing near the ends of long bones, responsible for bone elongation and therefore linear growth. We evaluated microarray and RNA-Seq data (13, 14) in mouse growth plates (and other tissues) focusing on three comparisons. First, we compared 1-week old whole growth plate tissues with three soft tissues (lung, kidney, and heart) to compare expression of each gene of interest in the growth plate to expression in other tissues (13). Second, we compared expression in three different zones: the resting, proliferative, and hypertrophic zone in 1-week old mice by RNA-seq. We asked whether a gene showed a spatial gene expression pattern suggesting functional importance for stem cell maintenance (resting zone), cell division (proliferative zone), or terminal differentiation (hypertrophic zone). Third, we compared 1- versus 4-week old proliferative and hypertrophic zones using RNAseq (14), asking whether a gene showed a temporal decline or increase in gene expression that would suggest regulation of proliferation or differentiation, as growth slows with age.

Our analysis showed that *Ift140* and *Nav2* were well expressed in the growth plate, showing much higher levels compared to soft tissues analysed (**Table 3A**). *Ift140* and *Nav2*, and to a lesser extent *Scaf11*, also showed significant downregulation of gene expression from proliferative to hypertrophic zone at 1 week. *Ift140* with fold change of -0.86 and a *p*-value of 2.98E-06 as well as in particular, *Nav2* with a fold change of -2.78 and a *p*-value of 1.91E-62 showed high changes between proliferative compared to hypertrophic zone, suggesting that they might be involved in chondrocyte proliferation and/or hypertrophic differentiation (**Table 3B**). Interestingly, *Nav2* expression decreases in the proliferative zone from 1- to 4-week, further suggesting that *Nav2* may act as an important regulator of chondrocyte proliferation (**Table 3C**).

**Table 3A:** Expression in the murine growth plate. (A) Comparison with three soft tissues.

Gene symbol	Growth Plate vs Heart		Growth Plate vs Kidney		Growth Plate vs Lung	
	Difference	p-value	Difference	p-value	Difference	p-value
*Ift140*	1.73	1.01E-05	1.27	0.01	1.51	2.0E-04
*Nav2*	1.03	0.77	1.38	0.01	1.34	0.01
*Hsd3b2*	-1.51	0.02	-4.17	5.40E-08	-1.195	0.25

mRNA expression was measured by microarray (Lui et al, 2012). Differences between tissues are expressed as log_2_(fold-difference). Positive values indicate greater expression in growth plate than in the comparator tissue; negative values indicate greater expression in the comparator tissue than in the growth plate. CNGB1, GGTLC2, SCAF11 had no annotated probes in this microarray dataset.

**Table 3B:** Spatial comparison – expression in mouse growth plates.

Gene symbol	Resting to Proliferative		Proliferative to Hypertrophic	
	Difference	p-value	Difference	p-value
*Ift140*	-0.34	0.18	-0.86	2.98E-06
***Nav2***	-0.35	0.13	-2.78	**1.91E-62**
*Scaf11*	-0.04	0.81	-0.23	6.0E-02
*Hsd3b2*	-0.60	NA	-0.36	0.93
*Cngb1*	0.35	0.88	-0.19	0.89

mRNA expression was measured by RNA-seq. Differences between tissues are expressed as log_2_(fold-difference). Positive values indicate greater expression in proliferative zone than resting zone or in the hypertrophic zone than proliferative zone; negative values indicate lower expression in proliferative zone than resting zone or in the hypertrophic zone than proliferative zone. GGTLC2 showed minimal expression.The bold values highlight highly significant values in NAV2.

**Table 3C:** Temporal comparison - expression in mouse growth plates.

Gene symbol	Proliferative 1wk vs 4wk		Hypertrophic 1wk vs 4wk	
	Difference	p-value	Difference	p-value
*Ift140*	0.16	0.87	0.29	0.66
***Nav2***	-1.05	**0.003**	0.05	0.95
*Scaf11*	-0.12	0.54	0.23	0.10
*Hsd3b2*	0.16	NA	0	NA
*Cngb1*	1.04	0.54	0.59	0.72

mRNA expression was measured by RNA-seq. Differences between tissues are expressed as log_2_(fold-difference). Positive values indicate increasing expression with age; negative values indicate decreasing expression. GGTLC2 showed minimal expression; wk, weeks. NA, in DESeq2 assigns a p-value of NA to genes containing count outliers, as identified using Cook's distance.The bold values highlight highly significant values in NAV2.

In summary, our expression data are consistent with a functional role of both *Ift140* and *Nav2* in the growth plate, with *Nav2* being the strongest of the two.

### Knockout of the *NAV2* ortholog in *Xenopus* supports a developmental role for body size

**Figure 3:**
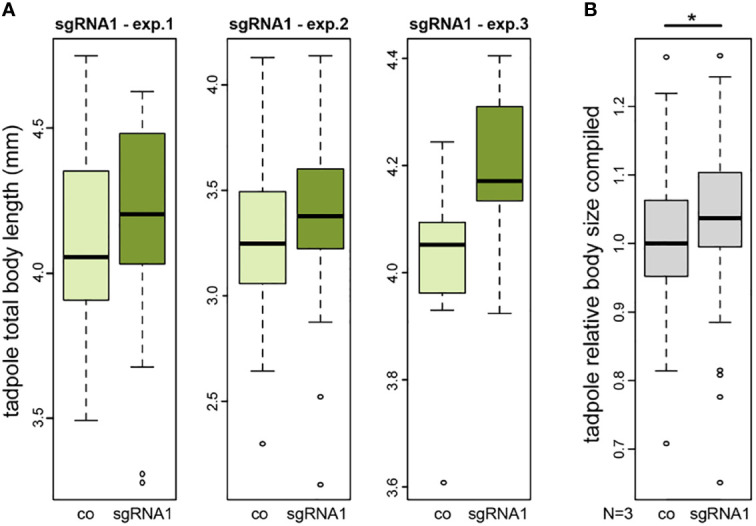
CRISPR/Cas-mediated knockout of *nav2* causes enhanced embryonic growth. **(A, B)** Tadpole total body length measurements of three independent experiments. All experiments revealed a slightly increased average body size in *nav2* sgRNA-injected specimens as compared to control littermates. **(A)** Three single experiments with sgRNA1 that specifically targets an exon orthologous to the exon harboring the human *NAV2* missense mutation p.R2201C (exp.1, n=38 animals, stage 45; exp.2, n=102 animals, stage 44; exp.3, n=18 animals, stage 45); **(B)** Combined data of the three experiments in **(A)** after normalization to the mean of their controls revealed a significant increase of relative body sizes after sgRNA1 injections (p-value=0.023). Boxplot whiskers (error bars) are 1.5 * IQR +Q_3_ (upper), or 1.5 * IQR -Q_1_ (lower). co, control; exp., experiment; sgRNA, single-guide RNA; mm, millimeter.

To functionally analyse *NAV2* in an animal model, we used a CRISPR/Cas9-based knockout approach in the African clawed frog *Xenopus laevis*. Due to restrictions by animal protection, we could only raise tadpoles until stage 45, i.e. about 5 days after fertilization. Thus, we could test for a developmental function of the *Xenopus nav2* gene during early body growth. To target the highly conserved exon harbouring the human arginine to cysteine missense mutation (R2142C), a single-guide RNA (sgRNA1) was designed. Injection of this sgRNA1 should generate a nonsense mutation or frameshift, leading to a truncated protein lacking the last six exons of the frog Nav2 protein, or alternatively, result in nonsense-mediated decay of the mRNA. We performed three injection experiments, injecting one-cell stage embryos and raised them until tadpole stage to quantify body sizes. Successful gene editing of injected specimens was evaluated by target sequence amplification and Sanger sequencing, demonstrating mutagenesis in about 35% of gene copies (for details, see Method section). Total tadpole body length of injected embryos showed increased body sizes in all three experiments (Exp1: +0.97%, Exp2: +3.36%, Exp3: +4.23%) (**Figure 3**). As the expected size differences were small and wild type body sizes differ naturally from batch to batch due to minor age differences, we could only compare between mutant and the corresponding wildtype. To be able to combine the data of the three different clutches, we normalized body lengths of each experiment to its controls. Statistical analysis of the combination of relative body sizes showed a significant increase after nav2 gene editing.

We also designed a second sgRNA2 targeting a large 5’ exon, to cause a complete nav2 gene knockout (see Methods, for details). Sequencing confirmed a mutagenesis rate of 75-93%. Again, no early phenotype was observed in two different experiments, but when raised to tadpole stage 45, about 50% of injected specimens started to develop organ defects and edemas from stage 42 onwards (**Supplementary Figures 2A–C**). The massive body defects resulted in general body size reduction, potentially overruling any increase in length (**Supplementary Figure 2D**). Yet, when we compared the other 50%, crispants without gross defects, we also found mildly but not statistically significant increase in body sizes, as previously in the knockout approach with sgRNA1 (Exp4: +0.47%, Exp5: +1.49%) (**Supplementary Figure 2E**).

Taken together, our data on nav2 mutants provide independent functional verification of mildly increased body size of 1-4% in five different experiments already at early stages of tadpole development.

### Two unrelated tall individuals with NAV2 variants

After we identified the candidate genes in this family, we re-examined exome sequencing data from a previously reported Dutch family with tall stature (12) and identified a rare *NAV2* variant in two family members, but not in the other candidate genes of the current study. This *NAV2* variant [c.G6112A, p.(E2038K)] was predicted as probably damaging by PPH2, damaging by Provean, deleterious by Sift and disease-causing by Mutation taster, with a CADD score of 26.8. The affected variant was conserved between human, mouse, chicken and Xenopus.

The variant [c.G6112A, p.(E2038K)] was identified in the two tallest family members, the index case (height 3.5 SDS) and his father (height 3.2. SDS). The variant was not carried by the sister of the index case (height 2.6 SDS) nor in the paternal uncle (height 2.9 SDS). All tall family members, however, shared damaging heterozygous variants in five different genes. This provides further evidence that damaging *NAV2* variants, in conjunction with other genes, can be associated with tall stature phenotypes.

## Discussion

In contrast to the success at genomic variant detection, the ability to distinguish pathogenic from benign missense variants by computational and experimental approaches is still challenging. Assessments of such variants, in particular missense variants, relies mostly on computational algorithms based on conservation. We have used a combinatorial approach to identify variants in new candidate genes for stature and classified the pathogenicity of these variants. This analysis enabled the identification of six candidate gene variants with a spectrum of likelihood of causality that were shared between all the genetically related family members with tall stature. The variants were not shared with the genetically unrelated spouse who married into this family, providing further evidence of a genetically heterogenous phenotype. Further evaluation of these six genes provided additional support on three of those genes (*IFT140, NAV2* and *SCAF11)* with the highest likelihood of having an effect fo*r IFT140* and in particular *NAV2* by expression analysis, GWAS studies on height, network analysis and growth plate expression analysis. Damaging *NAV2* variants (c.G6112A, p.E2038K) were also identified in two further unrelated tall individuals reported recently (12). Phenotype analysis of nav2 mutants in *Xenopus* provided further functional support.

*SCAF11* is expressed in many tissues, and diseases associated with SCAF11 include Corneal Endothelial Dystrophy as well as various types of cancer. Among its related pathways is the TNFR1 Pathway. Protein-protein interactions exist between SCAF11 and the transcription factor TCF7 and between SCAF11 and YWHAE, involved in signal transduction (24, 26). SCAF11 also interacts with the estrogen receptor ESR1 which itself is very highly associated with height (5.58E-87) (25). It is therefore tempting to speculate that the detected variant p.R983Q interferes with normal interaction of SCAF11 and ESR1.

Missense variants in *IFT140* have been reported to lead to “short-rib thoracic dysplasia 9 with or without polydactyly” (SRTD9, MIM #266920), also known as Mainzer-Saldino syndrome or retinitis pigmentosa 80 (RP80, MIM #617781) (30). Common signs and symptoms include a small chest and short ribs which restrict the growth and thorax expansion. The missense variant causing R1433C that we identified is monoallelic (biallelic in SRTD9) and resides in a different region at the 3´end of the *IFT140* gene. In N-ethyl-N-nitrosourea (ENU) induced mutagenesis, *Ift140* mutations have been shown to be associated with skeletal abnormality in mice, including craniofacial malformation, hindlimb polydactyly and forelimb poly- and oligodactyly (31). Molecular analysis of Ift140 cauli/cauli limb buds revealed altered spatial patterning and regulation of multiple growth factors, predominantly those in the cilia-dependent hedgehog signalling pathways. Indian hedgehog is an important factor in regulation within the growth plate, essential in the conversion from prehypertrophic to hypertrophic chondrocytes in the growth plate. Another interesting finding is that cilia play an important role in hedgehog signalling. Evidence that ciliary gene variants contribute to familial tall stature has been previously highlighted by our group (12). The ciliary protein IFT140 also interacts with the ciliary protein NEK2, and it is interesting that its homolog, *NEK1*, was previously identified as a gene underlying tall stature (12). Provided that the *IFT140* variant would have a gain of function, it could increase growth by slowing down the maturation of the epiphyseal chondrocytes. We therefore consider *IGF140* a reasonably strong candidate gene, but the evidence for *IFT140* is not as strong as for *NAV2*. Two or three knockout strategies at the same time are technical difficult and possibly even lethal in Xenopus, therefore we chose the best candidate, *NAV2*, for the functional studies.

Like *SCAF11* and *IFT140*, *NAV2* is expressed ubiquitously. It encodes a member of a gene family involved in cellular growth and cytoskeletal dynamics. Multiple transcript variants encoding different isoforms have been found for this gene. The affected amino acid in NAV2 is conserved to Drosophila, supporting strong biological significance. Nav2 function has been addressed in mammalian nervous system development, and shown to be required for normal cranial nerve development and also blood pressure regulation. Biallelic truncating variants in *NAV2* have been associated with a neurodevelopmental phenotype (32). *NAV2* is a vitamin A metabolite responsive gene and numerous cellular functions, including bone cell functions, are mediated by vitamin A (33). Vitamin A (retinol, a precursor of retinoic acid) cannot only stimulate the formation of bone-resorbing osteoclasts but also inhibit their formation (34). It has been shown that retinoic acid negatively regulates longitudinal bone growth by inhibiting growth plate chondrocyte proliferation, chondrocyte hypertrophy, and matrix synthesis (35). NAV2 positively modulates inflammatory response of fibroblast-like synoviocytes through activating Wnt/β-catenin signalling pathway in rheumatoid arthritis, which is also an important pathway in chondrocyte biology (36). NAV2 also interacts directly with several protooncogenes, like KRAS and HRAS, and the multiprotein complex “mediator”, also termed TRAP, which is highly associated with height (1.8E-26) (28). *Nav2* expression showed high changes between proliferative compared to hypertrophic zone, suggesting *Nav2* may act as an important regulator of chondrocyte proliferation.

Collectively, our analysis revealed several novel high-confidence candidate genes associated with isolated tall stature. A growing number of genes with both a gain of function and loss of function mutations associated with opposing effects on the phenotype are known in the literature (20). Opposing skeletal phenotypes (tall and short stature) resulting from pathogenic heterozygous variants in the same gene are highly suggestive of shared pathophysiology. Evidence has been provided, for example in *IGF1R, NPPC, NPR2, FGFR3*, as well as *FBN1* and *FBN2* for a bidirectional effect on height [summarized in (20)]. While *FGFR3* gain of function mutations lead to short stature (achondroplasia, ACH, MIM #100800 and hypochondroplasia, HCH, MIM #146000), *FGFR3* loss of function mutations cause camptodactyly, tall stature, and hearing loss syndrome (CATSHLS, MIM #610474) (37, 38). Loss of function mutations in the natriuretic peptide receptor B (NPR2) have been reported in causing acromesomelic dysplasia 1 (AMD1, MIM #602875) (39), while gain of function mutations in the same gene result in overgrowth disorder (5, 7). Gain of function effects of large translocations and duplications have been also described for NPPC, IGF1R and SHOX (20, 40).

Gain of function mutations, however, are hard to identify from *in silico* predictions and require functional validation. To develop this work further, we have assessed the effect of our strongest candidate by carrying out a Nav2 knockdown in the animal model *Xenopus laevis*. Our data show a small (1-4%) but consistent increase in body size already at early stages of tadpole development (stage 45) in all experiments, suggesting that our observation is the first sign of the beginning process of enhanced growth during post-metaphoric stages (41). The mild increase in body size was not sufficient to obtain statistical significance in the three single experiments, but sgRNA1 data combination after normalization of body sizes resulted in a significant average increase (**Figure 3**). Stage 45 in tadpoles corresponds approximately to the time around the date of birth of mouse or human. As an amphibian species, this animal model has some limitations which relates to the later larval development that takes more time and incorporates a striking change of anatomy when going through metamorphosis. Its unique advantages are, however, the well characterized external embryonic development, the high numbers of embryos available from one female and the large size of its oocytes which allows for manipulations in one- to 64-cell embryos (e.g. CRISPR/Cas9-mediated loss-of-function). On top of these benefits, as a basal tetrapod species, *Xenopus* genetics and physiology are still close enough to humans (42, 43).

Body height in particular is conferred by gene variants acting together, supporting an oligogenic inheritance. For these reasons we anticipate and cannot rule out that further gene variants that did not quite meet our selection criteria may also contribute to the phenotype by an accumulation of small or tiny effects, as rare gene variants with stronger effect and risk factors with minor effect likely work together.

## Data availability statement

The data presented in the study are deposited in the LOVD repository, variant numbers 0000921484, 0000921485, 0000921486, 0000921487, 0000921488, 0000921489, 0000921490.

## Ethics statement

The studies involving humans were approved by the research protocol (P06.118) “Genetic diagnostics of very tall stature”, Leiden University Medical Center. The study was also part of the Research Program “Whole exome genetic analyses in rare hereditary diseases” at Heidelberg University. The study was conducted in accordance with the guidelines of the WMA Declaration of Helsinki and the Department of Health and Human Services Belmont Report. The studies were conducted in accordance with the local legislation and institutional requirements. The participants provided their written informed consent to participate in this study. Written informed consent was obtained from the individual(s) for the publication of any potentially identifiable images or data included in this article. Handling, care and experimental manipulations of animals was approved by the Regional Government Stuttgart, Germany (V349/18 ZO) according to German regulations and laws (§6, article 1, sentence 2, nr. 4 of the animal protection act).

## Author contributions

BW: Methodology, Writing – review & editing, Data curation, Formal analysis, Investigation. TO: Writing – review & editing, Data curation, Formal analysis, Investigation. PV: Data curation, Formal analysis, Conceptualization, Methodology, Writing – original draft. JL: Data curation, Formal analysis, Methodology, Writing – review & editing. RR: Data curation, Writing – review & editing. SV: Data curation, Writing – review & editing. SW: Data curation, Writing – review & editing, Formal analysis, Methodology. SH: Formal analysis, Writing – review & editing. JB: Formal analysis, Writing – review & editing. JMW: Writing – review & editing, Conceptualization, Writing – original draft. GAR: Conceptualization, Writing – original draft, Writing – review & editing, Funding acquisition, Methodology, Supervision.

## Supplementary material

Supplementary Table 1 Primer.

Supplementary Table 2 GTEX data.

Supplementary Table 3 GWAS on height.

Supplementary Table 4 List of associated tall stature genes.

Supplementary Figure 1 Ingenuity (IPA) Network analysis. Known genes from the literature (n=86; in green colour) underlying tall stature were analysed using the IPA network analysis. The newly identified genes were added to predict functional connections in the context of known protein networks.

Supplementary Figure 2
*nav2* sgRNA2 causes wild type appearance in about half of the animals and organ defects and reduced embryonic growth in the other half. **(A–C)** Overall appearance of representative stage 45 control specimen **(A, A’)**, or *nav2* sgRNA2-injected specimen **(B, B’)**, illustrating organ malformations with edema formation in about 50% of *nav2* sgRNA2 injections. Tadpoles are shown in lateral **(A, B)** and ventral view **(A’, B’)**. **(C, D)** Quantification of total body length of tadpoles with organ defects (grey fraction in C), showing reduced body length when compared to control specimens **(A, C)**. **(E)** Quantification of total body length of tadpoles without organ defects (dark blue fraction in C) of the two individual experiments with sgRNA2 (exp.1, n=53 animals, stage 45; exp.2, n=31 animals, stage 45) demonstrating a mild but not statistically significant increase in body size. co, control; sgRNA, single-guide RNA; mm, millimeter; OD, organ defects; st., stage; wta, wild type appearance.
