# Supplementary material for: Identification of novel genes including NAV2 associated with isolated tall stature
Source: Front Endocrinol (Lausanne). 2023 Dec 12;14:1258313. doi: 10.3389/fendo.2023.1258313 (PMC10752378; doi:10.3389/fendo.2023.1258313)
Supplement: Supplementary file 1 [file DataSheet_1.docx]

**Supplementary information**

**Subjects and growth measurements**

The analysis of height in a multigenerational family is complicated because of three factors. First, accurate information about height in young adulthood is often unavailable, particularly in females. In most males of >50 years, a height measurement was performed at conscription, but at that age (18 years) mean height in the population is still 2 cm below height at 21 years (1,2), and more in case of delayed pubertal maturation. Second, an individual’s height decreases by age (“shrinking”), but there is variation between individuals regarding speed and amount of shrinking (3). Therefore, in a certain adult one can just assume that he or she has shrinked according to the mean pattern for sex observed in a population study (3). In a previous paper, we presented a mathematical equation to calculate height (cm) at 21 years based on current age (4). Third, in most West-European countries average adult height has considerably increased (positive secular trend), and this has been particularly strong in the Netherlands between 1850 until 1997 (1). However, the most recent population study showed no further increase (2).

Thus, for the calculation of adjusted adult height SDS, the estimated height at 21 years of age has to be compared with the population’s mean (SD) adult height in that particular year, derived from the regression lines for secular trend in the Netherlands (4). In generations I and II of the family presented in this paper we took these issues into consideration to generate a best estimate of the individual’s height SDS at 21 years of age. Height SDS of the two individuals in generation III were calculated on current nationwide references (2), which is virtually identical to the 1997 reference data (1). Head circumference SDS and sitting height/height SDS were calculated according to Dutch population references (1,5). Arm span/height SDS in children was calculated based on Dutch reference data (6). Arm span/height SDS in adults was based on the mean for age and sex in European adults (7) and the SD that we observed in 17-year olds (6). Physical examination of individuals II.1, II.2, II.4 and III.1 by two pediatric endocrinologists (courtesy Dr. Gerdine A Kamp and Dr. Hester Havers) did not show any unusual clinical features.

For the two individuals in generation I, we searched for information about height in young adulthood and their current height was measured by a relative. Case I.1, born in 1937, reported a height of 202 cm when measured as a conscript, which is equivalent to 3.6 SDS for reference data in 1958 (assuming no further growth between 18 and 21 years). Height in 2021 was 198 cm, which translates to 206.1 cm (4.2 SDS) if adjusted for mean shrinking for age. We chose 3.6 SDS as most likely.

Case I.2 (born in 1939) was measured in 2021 (176 cm). Assuming mean shrinking for age, her height at 21 years of life would have been 183 cm (2.8 SDS for 1960 reference data). However, according to her daughter, she had a height of 178 as a young woman, which would be 2.0 SDS for 1960 reference data. We consider the latter estimate most likely.

Case II.1, born in 1965, probably at term, with a weight of 3820 g (0.5 SDS) was extremely tall in adolescence and predicted to reach an adult height of 208-211 cm. He was treated with high dose testosterone injections for one year. He reported a height of 207 cm at 21 years of age (3.5 SDS for 1986 reference data), but at 56 years we measured his height at 208.6 cm, which would translate to 211 cm (4.0 SDS) at 21 years. We consider the latter most likely, although this may still be an underestimation in view of the treatment with testosterone esters in adolescence. Sitting height was 104 cm (sitting height/height -1.0 SDS) and head circumference 60 cm (1.3 SDS).

His spouse (II.2), born in 1964, had at one month of age a weight of 4.05 kg (0.1 SDS) and length of 57 cm (1.3 SDS). She reported a height of 189 cm as a young woman (adjusted height 3.0 SDS). At examination at 57 years she was 186.7 cm. Assuming mean shrinking, her height at 21 years was estimated at 190.6 cm (3.2 SDS). We consider the latter most likely. Sitting height/height ratio was -0.83 SDS, arm span/height -1.7 SDS, and head circumference 57 cm (1.0 SDS).

Their son (III.1), born in 2007, was born with a length of 54 cm (1.8 SDS) and weight of 4.12 kg (1.2 SDS). Head circumference at 6 weeks of age was 38.6 cm (0.9 SDS). During follow up at preventive health care, length at 1 years was 2.5 SDS, and height at 3 and 6 years was 1.7 and 2.3 SDS, respectively. The following years height remained between 2.0-2.5 SDS. He was first examined by a pediatrician at 13.6 years of age. His height was 183.6 (2.2 SDS). Sitting height was 92 cm (sitting height/height ratio -0.6 SDS). Arm span was 179.2 cm, resulting in an arm span/height ratio of 0.9760 (-1.6 SDS). Head circumference was 56 cm (0.6 SDS). Tanner stages were G3/P3, testicular volume 11/13 ml (Prader orchidometer). Bone age was 13-13.5 years, and predicted adult height according to Bayley-Pinneau was 203-206 cm (2.7-3.1 SDS). At 15.0 years his height was 198.1 cm (2.9 SDS) and predicted adult height was 204 cm (2.8 SDS). No unusual clinical features were observed. We chose the predicted adult height (2.8 SDS) as a proxy for adult height.

Case II.4 (born in 1963) reported that she had been treated with estrogens at the age of 12 years because of tall stature and a predicted adult height of 190 cm. We examined her at 57.6 years, when height was 183.3 cm. Adjusted for mean age-related shrinking, the estimated height at 21 years is 187.2 cm (2.7 SDS for the 1984 reference), but this is probably lower than adult height had been if no estrogens had been administered. We prefer to consider the predicted adult height before estrogen treatment as a better indicator of her biologically determined adult height (190 cm, equivalent to 3.2 SDS for the 1984 reference). Sitting height was 94.6 cm (sitting height/height ratio -0.7 SDS). Arm span was 176.6 cm (arm span/height ratio -2.3 SDS). Head circumference was 60.0 cm (2.8 SDS).

Her husband (II.3), born in 1962, reported a height of 190 cm at 59 years. Adjusted for mean shrinking, height at 21 years would have been 193.8 cm (1.7 SDS for the 1983 reference).

Their 23 years old daughter (III.2, born in 1998) reported a height of 166 cm (-0.7 SDS).

1. Fredriks AM, Van Buuren S, Burgmeijer RJ, Meulmeester JF, Beuker RJ, Brugman E, Roede MJ, Verloove-Vanhorick SP, Wit JM. Continuing positive secular growth change in The Netherlands 1955-1997. Pediatr Res. 2000;47(3):316-23.

2. Schonbeck Y, Talma H, Van Dommelen P, Bakker B, Buitendijk SE, Hirasing RA, Van Buuren S. The world's tallest nation has stopped growing taller: the height of Dutch children from 1955 to 2009. Pediatr Res. 2013;73(3):371-7.

3. Sorkin JD, Muller DC, Andres R. Longitudinal change in height of men and women: implications for interpretation of the body mass index: the Baltimore Longitudinal Study of Aging. Am J Epidemiol. 1999;150(9):969-77.

4. Niewenweg R, Smit ML, Walenkamp MJ, Wit JM. Adult height corrected for shrinking and secular trend. Ann Hum Biol. 2003;30(5):563-9.

5. Fredriks AM, Van Buuren S, van Heel WJ, Dijkman-Neerincx RH, Verloove-Vanhorick SP, Wit JM. Nationwide age references for sitting height, leg length, and sitting height/height ratio, and their diagnostic value for disproportionate growth disorders. Arch Dis Child. 2005;90(8):807-12.

6. Gerver WJM, Gkourogianni A, Dauber A, Nilsson O, Wit JM. Arm Span and Its Relation to Height in a 2- to 17-Year-Old Reference Population and Heterozygous Carriers of ACAN Variants. Horm Res Paediatr. 2020;93(3):164-72.

7. Quanjer PH, Capderou A, Mazicioglu MM, Aggarwal AN, Banik SD, Popovic S, Tayie FA, Golshan M, Ip MS, Zelter M. All-age relationship between arm span and height in different ethnic groups. Eur Respir J. 2014;44(4):905-12.
