# Supplementary material for: Identification of novel genes including NAV2 associated with isolated tall stature
Source: Front Endocrinol (Lausanne). 2023 Dec 12;14:1258313. doi: 10.3389/fendo.2023.1258313 (PMC10752378; doi:10.3389/fendo.2023.1258313)
Supplement: Supplementary Table 4 — List of associated tall stature genes. [file Table_4.docx]

| **Symbol** | **Name** |
| --- | --- |
| ***ABAT*** | 4-aminobutyrate aminotransferase |
| ***ACTA1*** | actin, alpha 1, skeletal muscle |
| ***ADAMTSL4*** | ADAMTS-like 4 |
| ***AGGF1*** | angiogenic factor with G patch and FHA domains 1 |
| ***AGPAT2*** | 1-acylglycerol-3-phosphate O-acyltransferase 2 |
| ***AIP*** | aryl hydrocarbon receptor interacting protein |
| ***AKT1*** | v-akt murine thymoma viral oncogene homolog 1 |
| ***AR*** | androgen receptor |
| ***BSCL2*** | Berardinelli-Seip congenital lipodystrophy 2 (seipin) |
| ***CBS*** | cystathionine-beta-synthase |
| ***CDKN1C*** | cyclin-dependent kinase inhibitor 1C (p57, Kip2) |
| ***CEP104*** | centrosomal protein 104 |
| ***COL2A1*** | collagen, type II, alpha 1 |
| ***COL6A1*** | collagen, type VI, alpha 1 |
| ***COL6A2*** | collagen, type VI, alpha 2 |
| ***COL6A3*** | collagen, type VI, alpha 3 |
| ***CROCC*** | rootletin |
| ***DIS3L2*** | DIS3 like 3'-5' exoribonuclease 2 |
| ***DNA2*** | DNA replication helicase/nuclease 2 |
| ***EFEMP2*** | EGF containing fibulin-like extracellular matrix protein 2 |
| ***EZH2*** | enhancer of zeste 2 polycomb repressive complex 2 subunit |
| ***FAM20A*** | family with sequence similarity 20, member A |
| ***FBN1*** | fibrillin 1 |
| ***FBN2*** | fibrillin 2 |
| ***FGF3*** | fibroblast growth factor 3 |
| ***FGFR3*** | fibroblast growth factor receptor 3 |
| ***GH1*** | growth hormone 1 |
| ***GLI3*** | GLI family zinc finger 3 |
| ***GNAS*** | GNAS complex locus |
| ***GPC3*** | glypican 3 |
| ***GPC4*** | glypican 4 |
| ***GRIA3*** | glutamate receptor, ionotropic, AMPA 3 |
| ***H19*** | H19, imprinted maternally expressed transcript (non-protein coding) |
| ***HPGD*** | hydroxyprostaglandin dehydrogenase 15-(NAD) |
| ***HRAS*** | Harvey rat sarcoma viral oncogene homolog |
| ***HSPG2*** | heparan sulfate proteoglycan 2 |
| ***INSR*** | insulin receptor |
| ***KCNQ1OT1*** | KCNQ1 opposite strand/antisense transcript 1 (non-protein coding) |
| ***KIF7*** | kinesin family member 7 |
| ***KRAS*** | Kirsten rat sarcoma viral oncogene homolog |
| ***LETM1*** | leucine zipper-EF-hand containing transmembrane protein 1 |
| ***LHCGR*** | luteinizing hormone/choriogonadotropin receptor |
| ***LRP4*** | low density lipoprotein receptor-related protein 4 |
| ***MC2R*** | melanocortin 2 receptor (adrenocorticotropic hormone) |
| ***MED12*** | mediator complex subunit 12 |
| ***MGAT2*** | mannosyl (alpha-1,6-)-glycoprotein beta-1,2-N-acetylglucosaminyltransferase |
| ***MTM1*** | myotubularin 1 |
| ***NEB*** | nebulin |
| ***NEK1*** | serin/ threonine-protein kinase |
| ***NELFA*** | negative elongation factor complex member A |
| ***NF1*** | neurofibromin 1 |
| ***NFIX*** | nuclear factor I/X (CCAAT-binding transcription factor) |
| ***NPR2*** | Natriuretic peptide receptor 2 |
| ***NPR-C*** | natriuretic peptide receptor-c |
| ***NSD1*** | nuclear receptor binding SET domain protein 1 |
| ***NSDHL*** | NAD(P) dependent steroid dehydrogenase-like |
| ***PAFAH1B1*** | platelet-activating factor acetylhydrolase 1b, regulatory subunit 1 (45kDa) |
| ***PIGA*** | phosphatidylinositol glycan anchor biosynthesis, class A |
| ***PIGL*** | phosphatidylinositol glycan anchor biosynthesis, class L |
| ***PIK3CA*** | phosphatidylinositol-4,5-bisphosphate 3-kinase, catalytic subunit alpha |
| ***PLOD1*** | procollagen-lysine, 2-oxoglutarate 5-dioxygenase 1 |
| ***POF1B*** | premature ovarian failure, 1B |
| ***PRKAR1A*** | protein kinase, cAMP-dependent, regulatory, type I, alpha |
| ***PTCH1*** | patched 1 |
| ***PTEN*** | phosphatase and tensin homolog |
| ***RET*** | ret proto-oncogene |
| ***SCARF2*** | scavenger receptor class F, member 2 |
| ***SHANK3*** | SH3 and multiple ankyrin repeat domains 3 |
| ***SHOX*** | short stature homeobox |
| ***SKI*** | SKI proto-oncogene |
| ***SLC6A8*** | solute carrier family 6 (neurotransmitter transporter), member 8 |
| ***SMS*** | spermine synthase |
| ***SOST*** | sclerostin |
| ***SRY*** | sex determining region Y |
| ***TGFB1*** | transforming growth factor, beta 1 |
| ***TGFB2*** | transforming growth factor, beta 2 |
| ***TGFBR1*** | transforming growth factor, beta receptor 1 |
| ***TGFBR2*** | transforming growth factor, beta receptor II (70/80kDa) |
| ***TOM1L2*** | thiosulfate sulfurtransferase-like domain containing 2 |
| ***TRIM32*** | tripartite motif containing 32 |
| ***TSTD2*** | target of myb1-like 2 |
| ***UPF3B*** | UPF3 regulator of nonsense transcripts homolog B (yeast) |
| ***WHSC1*** | Wolf-Hirschhorn syndrome candidate 1 |
| ***YWHAE*** | tyrosine 3-monooxygenase/tryptophan 5-monooxygenase activation protein, epsilon |
| ***ZDHHC9*** | zinc finger, DHHC-type containing 9 |
| ***ZNF469*** | zinc finger protein 469 |

**Supplementary Table 4**: Genes associated with Tall Stature (n=86) : 77 genes associated with the tall stature phenotype by mapping known disease genes to disease phenotypes from the HPO Gene-Disease Associations dataset ([HPO Gene-Disease Associations](https://maayanlab.cloud/Harmonizome/dataset/HPO+Gene-Disease+Associations); Rouillard, 2016) <http://compbio.charite.de/hpoweb/showterm?id=HP:0000098>

The following genes were added to this list: *NPR2, NPR-C, GH1, SHOX, as well as CEP104, CROCC, NEK1, TOM1L2, TSTD2* (Weiss et al, 2021).
