# Supplementary figures and images for: Identification of novel genes including NAV2 associated with isolated tall stature
Source: Front Endocrinol (Lausanne). 2023 Dec 12;14:1258313. doi: 10.3389/fendo.2023.1258313 (PMC10752378; doi:10.3389/fendo.2023.1258313)

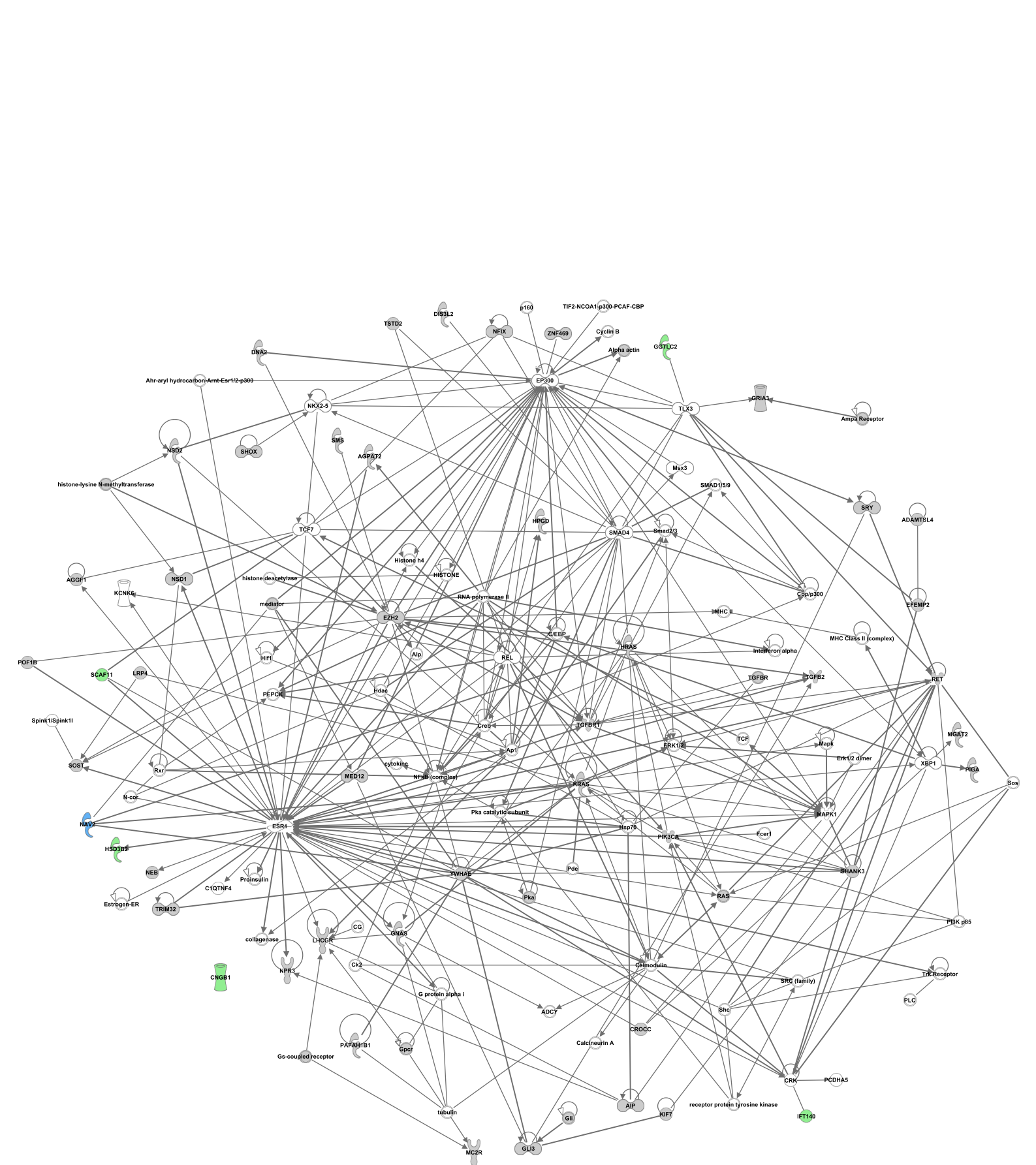

Supplement: Supplementary Figure 1 — Ingenuity (IPA) Network analysis. Known genes from the literature (n=86; in green colour) underlying tall stature were analysed using the IPA network analysis. The newly identified genes were added to predict functional connections in the context of known protein networks. [file Image_1.pdf]
