# Supplementary material for: Identification of novel genes including NAV2 associated with isolated tall stature
Source: Front Endocrinol (Lausanne). 2023 Dec 12;14:1258313. doi: 10.3389/fendo.2023.1258313 (PMC10752378; doi:10.3389/fendo.2023.1258313)
Supplement: Supplementary Figure 2 — nav2 sgRNA2 causes wild type appearance in about half of the animals and organ defects and reduced embryonic growth in the other half. (A–C) Overall appearance of representative stage 45 control specimen (A, A’), or nav2 sgRNA2-injected specimen (B, B’), illustrating organ malformations with edema formation in about 50% of nav2 sgRNA2 injections. Tadpoles are shown in lateral (A, B) and ventral view (A’, B’). (C, D) Quantification of total body length of tadpoles with organ defects (grey fraction in C), showing reduced body length when compared to control specimens (A, C). (E) Quantification of total body length of tadpoles without organ defects (dark blue fraction in C) of the two individual experiments with sgRNA2 (exp.1, n=53 animals, stage 45; exp.2, n=31 animals, stage 45) demonstrating a mild but not statistically significant increase in body size. co, control; sgRNA, single-guide RNA; mm, millimeter; OD, organ defects; st., stage; wta, wild type appearance. [file Image_2.pdf]

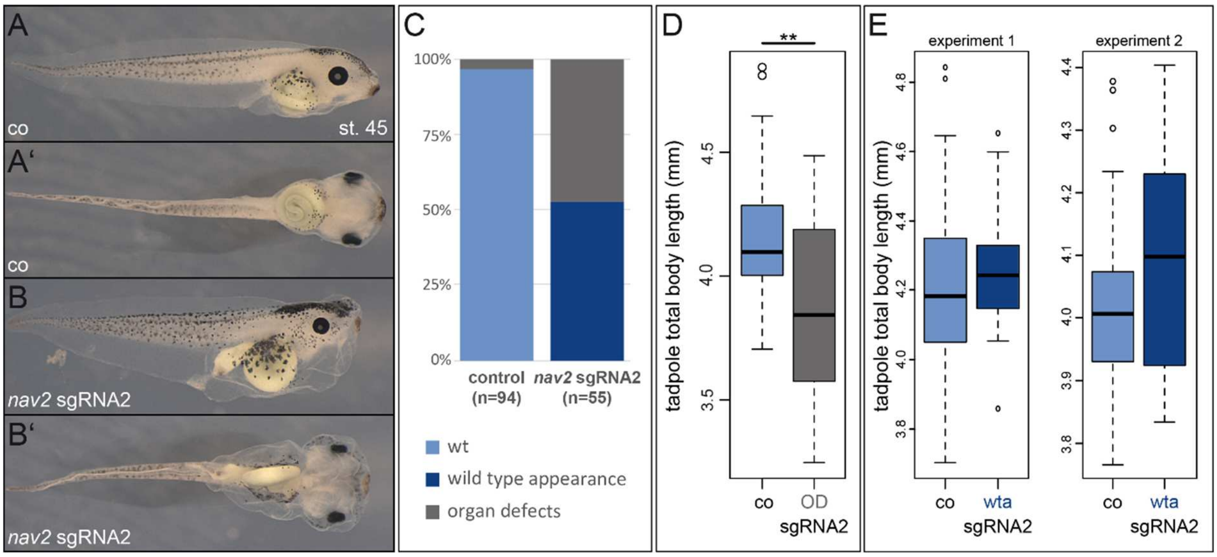

Supplementary Figure 2. *nav2* sgRNA2 causes wild type appearance in about half of the animals and organ defects and reduced embryonic growth in the other half. (**A-C**) Overall appearance of representative stage 45 control specimen (**A,A'**), or *nav2* sgRNA2-injected specimen (**B,B'**), illustrating organ malformations with edema formation in about 50% of *nav2* sgRNA2 injections. Tadpoles are shown in lateral (**A, B**) and ventral view (**A',B'**).(**C**,**D**) Quantification of total body length of tadpoles with organ defects (grey fraction in C), showing reduced body length when compared to control specimens (**A,C**). (**E**) Quantification of total body length of tadpoles without organ defects (dark blue fraction in C) of the two individual experiments with sgRNA2 (exp.1, n=53 animals, stage 45; exp.2, n=31 animals, stage 45) demonstrating a mild but not statistically significant increase in body sizes. co, control; sgRNA, single-guide RNA; mm, millimeter; OD, organ defects; st., stage; wta, wild type appearance.
